# Past-present-future childhoods: Technology, time, and childhoods in narratives of pandemic parenting

**DOI:** 10.1177/09075682251317131

**Published:** 2025-02-11

**Authors:** Lindsay C. Sheppard

**Affiliations:** 7991 York University, Canada

**Keywords:** Agential realism, childhood, parenting, technology, time

## Abstract

Re-turning interviews with 15 mothers in Southern Ontario about parenting during the COVID-19 pandemic, this paper explores meanings and experiences of childhood, children, and technology. Thinking with Karen Barad I ask: how is temporality evoked in stories of childhood and parenting in the context of the COVID-19 pandemic? Entangling with some of the material-discursive arrangements of childhood in the mothers’ narratives, I trace the differences that time, technology, and space enact for the boundaries of childhood. This theorizing can complicate conceptualizations of childhood, time, and linearity, by illustrating how past, present, and future childhoods are co-existing and co-constituting.

## Introduction

Multiple meanings and experiences of time affect how child and childhood are understood and embodied (James and Prout, 2015; Kohan, 2015; Murris, 2016). For example, how should time in childhood be filled? Is time experienced differently in adulthood? In Childhood Studies, social constructivist perspectives of time are prominent, recognizing time as a contextual social creation, for example characterizing the “time *of* childhood” as a discrete developmental stage, and the “time *in* childhood” where “time is used to produce, control and order the everyday lives of children” (James and Prout, 2015: 202). Further, “time passed” is evident in a focus on developmental age, and “future time” through a focus on children becoming adults (James and Prout, 2015). Time is also evoked when childhood is positioned as a period to look back on with nostalgia (James and Prout, 2015; Livingstone and Blum-Ross, 2020). Sometimes however, children and childhood are seen as outside of or detached from time, as idealized childhood innocence and naivety shield them from the pressure and presence of time as felt by adults (James and Prout, 2015). Enchanted by theorizing about temporality in Childhood Studies (e.g., Crinall, 2017; Crinall et al., 2020; James and Prout, 2015; Kohan, 2015; Murris, 2016), I diffract Barad’s (2007, 2014, 2017) agential realist account of temporality with mothers’ stories of parenting with technology to understand how meanings and experiences of time, child(hood), and adult(hood) emerge together.

Responding to the centring of discourse in analyses, there is a growing body of research that explores relational, new materialist, and posthuman approaches to childhood which aim to attend to the multiple and interconnected material things, discourses, times, and spaces which shape meanings and experiences of child and childhood (e.g., Crinall, 2017; Crinall et al., 2020; Murris, 2016; Murris and Kohan, 2021; Spyrou, 2019). Such approaches resist an ontological hierarchy and grapple with how multiple human and non-human forces collide with and co-create meanings and boundaries of child and childhood (Spyrou, 2019). My thinking about child and childhood are shaped by Murris’ (2016) *Posthuman Child.* Drawing on theorists like Barad, Braidotti, Deleuze, Haraway, and MacLure, Murris (2016) presents the figuration of the posthuman child as a relational “nomadic subject.” A posthuman child is not a pre-existing individual with discrete fleshy boundaries, rather an entanglement that becomes through relations of materialities-discourses-times-spaces (Murris, 2016). The human body child is not at the center of Murris’ (2016) posthuman child, rather child is an entanglement of multiple bodies, materialities, discourses, times, and spaces. Murris troubles the adult-child binary by advancing “childadult” as a non-dichotomous re-conceptualization that opens child and childhood up, “mak[ing] room for the not-yet-fully-human of humanism, that is, the ‘child’” (2016: 104). Murris’ childadult blurs boundaries between child-adult to challenge developmentalist, binary, and chronological conceptualizations of child(hood) which hinge on adult(hood). When child and adult are understood as material-discursive phenomena it is not possible to fix child and adult to discrete fleshy bodies or developmental/chronological times with beginnings and ends. Child and adult are entwined, co-constitutive material-discursive entanglements (Murris, 2016).

With Murris (2016) and Barad (2007, 2014, 2017) I trace difference in entanglements of materialities-discourses-times-spaces and their effects for co-constituting child(hood) and adult(hood). I re-turn (Barad, 2014) seven moments from Zoom interviews with mothers about parenting with technology during the COVID-19 pandemic, and diffract them with Barad’s agential realism (see also Pomerantz and Raby, 2018). In re-turning to the transcripts through a diffractive methodology, I “aerate” them, turn them over, continuously diffracting (Barad, 2014) them with theory. Thus, the interviews and transcripts are iteratively becoming. I was struck by the messiness and multiplicity of time in the stories, including how mothers evoked screen-time, pandemic time, “the time of childhood” (James and Prout, 2015), future time, and childhood nostalgia. I became curious about how parents’ thoughts about their children’s screen-time and technology use in the pandemic might provoke a rethinking of the linearity of childhood and adulthood. Re-turning to the data, I wondered: how is temporality evoked in stories of childhood and parenting in the context of the COVID-19 pandemic?

Through a diffractive reading where I read the data and theory with and through each other (Mazzei, 2014; Lenz Taguchi, 2012), I aim to “trouble time” (Barad, 2017) to illustrate the ways that adulthoods and childhoods of the past, present, and future are co-existing and co-constitutive, emergent through their entwinement (see also Crinall, 2017). An agential realist encounter of temporality and childhood is fruitful for recognizing child, childhood, adult, and adulthood as relational, multiple, and reconfiguring material-discursive entanglements (Murris, 2016). What follows is a diffractive reading of literature on screen time and technology during the COVID-19 pandemic, Barad’s agential realism, and interviews.

## Parenting and technology in the literature

Screen-time is prominently evoked in popular discourse, parenting advice, and research about children and childhood. For example, there is emphasis in parenting advice and research, for parents to monitor their children’s screen-time and encourage high-quality educational and developmentally appropriate technology (e.g., Ponti, 2023). While there are clear age-based recommendations for children’s screen-time (Ponti, 2023), researchers have argued that discussions of screen-time need to be nuanced, for example to think about different types of screen-time and the increasingly blurred boundaries of online and offline (Cowan and Potter, 2021; Livingstone and Blum-Ross, 2020; Toombs et al., 2022). Nuances of power and marginalization shape parenting practices and the possibilities for adhering to these screen-time recommendations, including access to technology and childcare, parental health and stress, as well as time and energy to monitor children’s technology use (Findley et al., 2022; Livingstone and Blum-Ross, 2020). Debates around screen-time, including whether children should engage with screens, at what age, and for how long illuminate how expectations and norms around the “time of childhood” shape how “time in childhood” is managed, affecting children’s lives (James and Prout, 2015).

Livingstone and Blum-Ross (2020) argue that technology is a key difference between parents’ childhoods and their children’s childhoods, and thus, dilemmas around technology elicit debates around values, identity, and responsibility. Parents evaluate themselves and other parents around how they manage their children’s screen-time, revealing the hyper-responsibility and individualism in narratives of “good” parenting (Findley et al., 2022; Livingstone and Blum-Ross, 2020; Willet and Zhao, 2024). Furthermore, expectations and discourses of parenting are shaped by intersections of gender, race, age, and social class, including in discourses of intensive mothering (Faircloth, 2014; Romagnoli and Wall, 2012). Parents’ actions and thoughts about children’s technology use are influenced by memories and expectations of the past, experiences in the present, and fears about the future (Livingstone and Blum-Ross, 2020; Rixon et al., 2019). Parents have reported that their present parenting around technology, such as which devices and software their children use, as well as screen-time rules, are shaped by their anxieties around fostering their children’s successful futures, including balancing the need for technological skill while also encouraging screen-free play and development (e.g., Livingstone and Blum-Ross, 2020). Additionally, childhood nostalgia can be evoked by material objects like media and technology, affecting parenting with technology (Livingstone and Blum-Ross, 2020; Wesseling, 2018). However, while reflecting on their childhoods, adults tend to reproduce idealized constructions of childhood which may not accurately represent their experiences, revealing the saliency of discourses of “good” parenting and childhood innocence (Malone, 2016; Rixon et al., 2019; Wesseling, 2018).

There is a growing body of quantitative and qualitative research on children and technology during the COVID-19 pandemic. For example, research indicates that many children's and youth’s screen-time increased during the pandemic (Findley et al., 2022; Hood et al., 2021; Toombs et al., 2022; Willet and Zhao, 2024). Researchers connect this increased screen-time to public-health mandates, disruptions in school, childcare, and extracurriculars, as well as parents’ work-from-home realities (Bergmann et al., 2022; Toombs et al., 2022). Increased parental stress during the COVID-19 pandemic was correlated with children’s increased screen-time (McArthur et al., 2021; Seguin et al., 2021). Additionally, families’ technology practices shifted during the COVID-19 pandemic, including, introducing new devices and software, and expanding digital play and communication (e.g., Cowan and Potter, 2021; Waboso et al., 2023). This research demonstrates how pandemic time shifted children’s screen-time, thus, affecting how children’s daily time was managed and filled during the pandemic.

In what follows, I add to this literature by considering how mothers’ stories on parenting with technology during the COVID-19 pandemic contain multiple times such as pandemic time, screen-time, past time, present time, and future time. I map the material-discursive entanglements in their stories and consider their effects for marking boundaries of child, childhood, adult, and adulthood. Overall, I aim to illustrate how multiple past-present-future childhoods and adulthoods are co-existing and co-constituting.

## Barad’s agential realism

My becoming with new materialist approaches began the summer before graduate school, when I read Barad’s (2007) *Meeting The Universe Halfway: Quantum Physics and the Entanglement of Matter and Meaning*. I continued to become with agential realism by reading Barad (2014), and later encountered Barad’s (2017) yearning to “trouble time” during the COVID-19 pandemic, when this research project began. Affected by Barad’s agential realist approach (2007), I continue to entwine messily with their theorizing even when a project starts from another theoretical or methodological position. Here, I move with and between these three texts in my diffractive analysis. I diffract more deeply with certain texts in specific moments, as the interview or text pulled me towards them. Recognizing the breadth of Barad’s theorizing, here, I focus on intra-activity, spacetimemattering, and self as they resonated most strongly in this research encounter.

Echoing Indigenous knowledge and scholarship (Todd, 2016), Barad critiques human exceptionalism and refutes a natural or cultural division between nature/culture, human/non-human, and material/discursive (2007). Barad proposes agential realism as an opportunity to explore material-discursive practices and how they come to matter, as well as patterns of difference (diffraction) that shape the co-constitution of reality (2007, 2014). Agential realism does not position phenomena as mental representations or discrete entities, rather as “entangled material agencies” (Barad, 2007: 56). Agency is not a characteristic or trait that can be given to humans or material things, rather intra-actions evoke an entanglement of multiple agencies (Barad, 2007). This means that matter is not fixed or the result of a process, rather “is produced and productive, generated and generative” (2007: 137). Mattering is a process which produces difference, as differentiating (Barad, 2007). One way to explore difference is through disentangling specific intra-actions (Barad, 2007).

Unlike interaction which suggests that materialities, discourses, time, and space have pre-existing meanings and boundaries which then produce effects, intra-action challenges the notion that things and meanings exist in an ontological hierarchy (2007). Instead, Barad’s intra-action positions matter and meaning as entities which emerge only through specific material-discursive entanglements (2007). Entanglements are specific configurations of materialities and discourses which co-create the possibilities for, and effects of the intra-action (Barad, 2007). Barad clarifies that entanglements do not necessarily change from one moment to the next, or across space, rather “space, time, and matter do not exist prior to the intra-actions that reconstitute entanglements” (2007: 74). Space and time are iteratively co-constituted through specific material-discursive entanglements in intra-activity (Barad, 2007, 2017). Barad uses spacetimemattering to position “spacetime [as] an enactment of differentness, a way of making/marking here and now” (2007: 137). This framing of spacetime recognizes that pasts, presents, and futures are porous, continuously becoming, and co-constitutive, intra-actively enfolded through each other (Barad, 2017). At the crux of Barad’s theorizing is exploring the specificity of material-discursive entanglements and intra-activity to understand how they produce difference, mark boundaries, and co-constitute what comes to matter (2007, 2014).

In addition to troubling binaries of nature/culture, materiality/discourse, and human/non-human, Barad challenges the self/other binary (2007). For Barad, the self is co-constituted by intra-activity, does not end at the boundaries of the human body, and is a multiplicity, “a superposition of beings, becomings, here and there’s, now and then’s” (2014: 176). Human and non-human bodies are thus always shaped by other human and non-human bodies, and multiple spacetimes, as meanings and boundaries are open and dynamic (Barad, 2014). The “I” is never complete and does not proceed intra-action, rather the “I” and the self emerge through intra-action, which in their repetition “are sedimented into our becoming, they become us” (Barad, 2007: 394). The self then, is not separate from the ongoing becoming and intra-activity of the world. This framing of self is important in the context of parenting and childhood, as it helps us to grapple with the entangled and co-constitutive material-discursive becoming of child and adult, including their force in the world’s ongoing intra-activity.

Barad’s agential realism opens opportunities for thinking about the material-discursive complexities and relational becomings of adult(hood) and child(hood) in particular spacetimes. Child(hood) and adult(hood) are not contained in fleshy bodies, rather are entanglements of bodies-materialities-discourses-spaces-time. Challenging figurations of child and adult tied to chronological age, an agential realist account provokes thinking about which spacetimematterings (Barad, 2007) mark boundaries of adult and child, and in turn, which adult(hood) and child(hood) bodies and subjectivities are enabled or constrained. Child(hood) and adult(hood) are “leaky” and “porous” (Barad, 2017), emergent through intra-activity wherein multiple past-present-future childhoods and adulthoods arise messily and at once. Echoing Murris (2016), I argue this re-conceptualization of child(hood) troubles linear understandings of time, and binaries of adult/child that marginalise and other child(hood). Below, I diffract Barad with mothers’ stories of pandemic parenting to unravel and entwine with the material-discursive contexts of childhood, parenting, technology, time, and space. I aim to underscore the impossibility of completely escaping child(hood) and progressing towards adult(hood) as traces of past-present-future childhoods and adulthoods intra-act in continual becoming.

## The research entanglement

I explore with data from a pilot project that I completed with Dr Natalie Coulter from January to July 2022. We worked collaboratively with researchers in Australia, China, Colombia, South Korea, the United Kingdom, and the United States, who all completed similar research projects (see Willet and Zhao, 2024). We met regularly on Zoom throughout the course of the COVID-19 pandemic to discuss findings from our research, and to brainstorm forthcoming writing projects. Our pilot study involved semi-structured Zoom interviews with 15 mothers in Southern Ontario about how the COVID-19 pandemic was shaping their families’ technology use. We asked about rules around technology and screen-time before and during the pandemic, the introduction of new devices and software, virtual schooling, and their aspirations for their families’ post-pandemic technology use. We were also curious about parents’ thoughts about children and technology more broadly, and whether their views were shifting over the course of the COVID-19 pandemic. We posted digital flyers in neighbourhood-specific parenting groups on Facebook in Southern Ontario. While we aimed to recruit parents and caregivers with children aged 4-12, only mothers expressed interest. Interviews lasted 60 minutes, were recorded, transcribed, and coded thematically using a list of codes and Dedoose qualitative software.

Based on participants’ self-identifications, the most common family structure in our pilot sample is heterosexual couples with two children. Although one participant was solo parenting while her partner was working overseas. Nearly all the interviewees had post-secondary education. We sought to understand participants’ social class by asking about their education, occupation, family structure, housing situation, and whether their children went to public or private school. Based on this information, three participants can be characterized as working class, nine as middle class, and three as upper-middle class. Notably, three of the mothers interviewed shared that they shifted their work during the pandemic for childcare or to support virtual schooling, including by retiring early, quitting their job, or switching to flexible or part-time hours. In their own words, eight participants self-identified as white, two as South Asian (Indian), two as Chinese-Canadian, one as Korean, and two self-identified as Chinese. While I discuss participants’ demographics here, I recognize how Barad (2007) troubles a centering of individual, contained human bodies as the sole sites of intersecting social factors like gender, race, sexuality, and social class. Instead, Barad (2007) argues that intertwined and intersecting forces of power and marginalization emerge through their intra-activity with other human and non-human bodies in specific material-discursive contexts. Such an approach to power and marginalization provokes thinking about what particular bodies, objects, times, and spaces *do* for constraining or enabling specific intra-actions (Barad, 2007), a point which I elaborate later.

## Diffractive methodology

The data I draw on in this paper was not created through a new materialist, posthuman or post-qualitative project. Importantly, the project began with a particular humanist, binary, linear, and age-based definition of child (i.e. 4-12 years old) and adult (i.e. parent/guardian). Our interview questions also included conceptualizations of time, for example when we asked about pre-pandemic, pandemic, and post-pandemic. Further, the interviews were semi-structured, recorded, and coded thematically, a process which some scholars argue resembles more traditional qualitative research processes, and thus, is seen as incompatible with new materialist and posthuman analysis (MacLure, 2013; Ringrose and Renold, 2014), and post-qualitative inquiry (St Pierre, 2019). While coding the data thematically helped me to make some sense of the data, I felt most compelled by the ways that nostalgia and time were weaved through many of the participants’ narratives. I recognize this as reflective of MacLure’s approach to wonder and sense (2013). MacLure (2013) encourages researchers to tune into sense as a research practice, arguing that “data have their ways of making themselves intelligible to us” through moments when we feel “especially ‘interested’ in a piece of data” (660). The wondrous data around temporality and nostalgia inspired this paper. I was curious how a diffractive reading and an agential realist approach to temporality might trouble humanist, binary, and linear conceptualizations of child, childhood, adult, and adulthood, illustrating how they iteratively unfold and entwine together (see also Crinall, 2017).

Moved by my curiosity about time and nostalgia in the mothers’ narratives of pandemic parenting, I re-read the data diffractively (Barad, 2007). Inspired by Haraway’s (1992) diffraction, Barad’s (2007) diffraction attends to patterns of difference and their effects. Barad (2007) uses a wave imagery to explain that as a rock in the ocean shifts the resulting wave patterns, different theories, data, methods, and questions collide with each other, shaping the emergent knowledge. Employing a diffractive methodology requires attention to embeddedness and relationality, and the ways that knowledge is always created intra-actively, within a particular research entanglement (Barad, 2007).

A diffractive methodology moves away from representational knowledge, towards difference and its effects. In practice, diffractive methodology can include reading data and theory through each other, and asking “what does this text or data produce?” rather than “what does it mean?” (Lenz Taguchi, 2012: 268). As Barad explains (2017: 64) “diffraction as a methodology is a matter of reading insights through rather than against each other to make evident the always-already entanglement of specific ideas in their materiality.” Through this methodology, the goal is not “to make analogies, but rather to explore patterns of difference…differentiating” (Barad, 2017: 64-65). Mazzei (2014) and MacLure (2013) argue that this shift away from meaning towards “doing” avoids an “easy sense” of the data, inviting researchers to engage with data that is particularly striking, troubling, or that which might resist analysis. Further, diffractive methodologies recognize research as intra-activity, as co-created through specific material-discursive entanglements that include multiple human and non-human bodies, technologies, times, and spaces (Barad, 2007). Thus, researchers are not outside of the intra-activity of research, and interviews are a specific spacetimemattering, a phenomena where matter and meaning are co-constitutive. In what follows, I narrow in on seven moments in the research entanglement that I found particularly wondrous (MacLure, 2013) and diffract them with Barad (2007, 2014, 2017) to explore how temporality is evoked in mothers’ stories on pandemic parenting. I disentangle and entangle with the interviews, aware that my embeddedness in the research entanglement as a non-parent, twenty-something graduate student researcher intra-act to co-create the story here. I attempt to move beyond a representational fixing of the data into neat themes or subthemes, and instead grapple with what the data *does* or produces for meanings and boundaries of child, childhood, adult, adulthood, parenting, and time.

### Diffraction one: Children-mother-phones-playground-old school child(hood)adult(hood)

We asked parents about their pre-pandemic thoughts about children and technology. We were curious about their rules and practices around screen-time and particular devices, but also about how they thought about parenting with technology more broadly. It was often in these moments where parents’ stories provoked me to think about Barad’s intra-activity in which spacetimematter are co-created (2017). When I asked Rose, whom had a daughter aged 9, to think back to her pre-pandemic thoughts about children and technology, she seemed overwhelmed with nostalgia. Rose was particularly worried about online safety, noting how she enthusiastically participates in online parenting groups about technology, and tries to stay informed about the latest parenting advice from “experts.” Indeed, like all the parents we spoke to, Rose shared how the pandemic undoubtedly affected her child’s technology use. When recalling pre-pandemic times Rose said, “I wanted her to have a child-like life like mine, where you just leave the house and play with the neighbour kids […] me and my husband did extremely well without all that [technology].”

During the interviews, we did a photo-elicitation activity where we found an online image using keywords that described participants’ thoughts about pandemic parenting. Rose selected an image where kids were gathered around a screen. Rose told me that this image was familiar to her, a scene she frequently observes at her daughter’s school playground, which she described as “heartbreaking.” While she reasoned that the children she observes gathered around phones at the playground are possibly watching TikTok videos, and maybe they are learning and connecting over the device, she seemed to yearn for a different kind of childhood for her daughter and her peers. Rose clarified that adults too gather over a screen in interaction with each other, but in grappling with her thoughts surrounding the photo she confessed, “I don’t know, I’m just old school.” I am affected by Rose’s use of “old school” here. I wonder, what material-discursive contexts enable becoming “old school” parent-child? Evoking “old school” parent(ing) and child(hood), Rose points to how matter (e.g., child bodies, parent bodies, phones, TikTok videos, playgrounds) and meaning (e.g., child, childhood, parent, adulthood) become legible through their co-creation. A diffractive reading of Rose’s narrative provokes me to think with Barad (2007, 2017) about the ways that Rose’s childhood, her husband’s childhood, their parenting, and their daughter’s childhood become with and through each other. As Barad (2007, 2017) argues, spacetime are made through intra-actions in specific entanglements, and do not exist prior to the intra-activity. Further, the boundaries of past-present-future are porous, as different spacetimes “bleed[] through one another” (Barad, 2017: 57). This orientation towards spacetime Barad argues (2017), means that time enacts difference in all becomings, as their effects are marked and preserved in the intra-activity that enacts difference in iterative becomings. Put differently, Rose’s childhood and her husband’s childhood leave a mark and are never complete or erased from the intra-activity which co-constitutes their parenting and their daughter’s childhood. Challenging a linear perspective of time and development, a diffractive reading acknowledges the ways that past-present-future childhoods are co-constituting, “living inside” each other (Barad, 2017: 57).

A diffractive reading also involves enacting an agential cut, which temporality isolates and disentangles the intertwinement of materialities and discourses to understand intra-activity (Barad, 2007). Rose’s narratives include material-discursive entanglements of play-time-space-technology-childhood. Her conceptualizations of technology and childhood are made through their intra-action, wherein childhood and technology become through their mutual co-constitution. Technology and childhood are intertwined materialities, such as cellphones and TikToks, and discursive, including “old school” and “child-like life.” These materialities and discourses cannot be understood outside of this specific entanglement. Rose’s understandings of childhood and technology are inseparable from the specific materialities (TikToks, cellphones, playground), discourses (“old school”, “child-like life”, “play”), time (Rose’s childhood, her daughter’s childhood), and space (playground) that shape and are shaped by each other. With Barad (2007), Rose’s narratives underscore the ways that materialities and discourses do not sit still, rather they *do* something for what comes to matter.

### Diffraction two: Pre-pandemic child(hood)-pandemic child(hood)-screentime

When asked about her pre-pandemic thoughts around children and technology, quite directly, Ruth said:

my thoughts were [to] keep them off screens and off video games and off social media for as long as humanly possible […] It’s like, it kills me with how pre-pandemic it was […] and how much like screen-time that they have managed to [accrue], like over the last two years, it is purely a result of the pandemic.

Ruth and her partner worked from home and their two children switched between online and in-person learning throughout the pandemic. I was struck by Ruth’s reflections while re-turning the data, particularly how meanings and experiences of child, childhood, and technology are entangled, emergent through their intra-action within the nuanced material-discursive context of the COVID-19 pandemic. Technology and screens like video games and social media take on specific meanings in their intra-action with the material-discursive contexts of childhood, in the spacetime (Barad, 2007) of the pandemic. Particular technologies like social media and video games are not merely material things, they are intertwined with discourses around child and childhood including screen-time and age, echoing findings from others (Livingstone and Blum-Ross, 2020). “Too much” screen-time emerges through an intra-action of pre-pandemic child(hood)-technology-timespace-pandemic child(hood), wherein entangled agencies mark their boundaries. Technology in the spacetimemattering (Barad, 2007) of the pandemic is not a neutral thing that pre-exists outside of or before Ruth’s meanings of child and childhood. Technology is also more than a socially constructed object. In the entanglement child-parent-technology-pandemic time-screentime-(pre)pandemic time, boundaries of “ideal” and “appropriate” child and childhood are marked. The timespace of the pandemic and the timespace of pre-pandemic differentiate Ruth’s conceptualizations of “ideal” and “not ideal” child body-technology-time-childhood relations. This differentiating troubles a neat fixing of child and childhood to a body and developmental time that are pre-existing and outside of the material-discursive world, rather recognizes them as material-discursive phenomena reconfigured in specific spacetimematterings (Barad, 2007).

A diffractive reading also challenges a linear framing of time, wherein childhood progresses forward. For Barad, the “new” and the “old” co-exist, “where one does not triumph by replacing and overcoming the other” (2017: 69). For Ruth, her children’s pre-pandemic technology use does not disappear becoming replaced by present technology use. The “old” child-parent-technology relations leave a mark, shaping the iterative unfolding and becoming of child and parent with and through technology. The past-present-future child, childhood, parent, parenting are enfolded through each other intra-actively. Erasure is never accomplished because it is always preserved in entanglements that enact difference in ongoing intra-actions (Barad, 2007). It is not possible to “move back” to pre-pandemic child-parent-technology relations as if they are unaffected by the timespacemattering (Barad, 2007) of the pandemic. The intra-actions that continuously co-constitute child bodies, parent bodies, and the material-discursive contexts of parenting and childhood contain traces of past-present-future. With Barad, I wonder what differences the timespacemattering of the COVID-19 pandemic do for the iterative becoming of child and childhood?

### Diffraction three: Child-childhood-money-phone-ego

When asked if she wanted to add anything to our conversation, Lady said:

something I’ve noticed amongst children, even when I was young, because when I was in grade 7 [or] 8 […] smaller flip phones were coming out. And like the cool kids, aka the rich kids, you know, had cellphones […] And not a lot has changed in that devices […] have become sort of a status symbol for children. And part of my aversion to [my daughter] having technology at a young age […] is to avoid that inflated ego.

Disentangling Lady’s story reveals how intertwined materialities of cellphones, intersections of age and class, and discourses of childhood enact difference in Lady’s emergent understanding of child, childhood, and pandemic parenting. Early in our interview, quite vulnerably, Lady shared how the pandemic was a difficult and precarious time, as her family moved in and out of shelters and had unstable Internet access which complicated virtual schooling. This moment “glowed” (MacLure, 2013) inspiring me to think about the ways that power and marginalization enact force in intra-activity. Material-discursive contexts reconfigure relations of power, which constrain and enable particular becomings of child, childhood, parent, and parenting (Barad, 2007). Re-turning (Barad, 2014) her childhood, Lady suggests becoming child with a flip phone materializes social class and differentiates the “cool” “rich” kids from those who are not. With Barad (2007), this entanglement of money-child-phone underscores how power and marginalization are emergent through intra-actions in specific material-discursive contexts, rather than contained in individual human bodies.

Diffracting Lady’s story opens possibilities for challenging a linear conceptualization of childhood and technology as “new” or remarkably “innovative.” As Barad (2017: 57) notes, in “troubling time” we can “undo pervasive conceptions of temporality that take progress as inevitable and the past as something that has passed and is no longer with us.” Like Rose, Lady’s story reveals how her own childhood is never finished, has left a mark, and continues to create difference in the intra-activity of her parenting and her daughter’s childhood. Put differently, her childhood, and some of its material-discursive contexts such as flip phones, exist in the intra-activity that shapes her meanings and experiences of parenting with technology. In addition, Lady reveals that “not a lot has changed,” which when read with Barad (2017) points towards the impossibility of absolute erasure, “newness,” or complete innovation, as past-present-future coexist and are enfolded through each other. An agential realist approach recognizes child and childhood as intra-active entanglements of bodies-spaces-times-discourses-materialities that are marked by material-discursive contexts of power and marginalization.

### Diffraction four: Past-present-future-child-parent-technology-screens-rules

Miriam, mother of a four-year-old boy, stopped working during the pandemic because of childcare closures. Throughout our interview, Miriam wondered whether her childhood experiences shaped rules about her son’s screen-time. Miriam said, “sometimes I think about [my childhood] and I probably just, like I don’t know what the rules were. I guess I should ask my parents […] I know, like I used to watch certain shows.” Diffracting Miriam’s story with Barad challenges a linear approach to temporality that disjoints child, parent, childhood, and adulthood (2014, 2017). I see Miriam’s reflections as re-turning, a “multiplicity of processes” not simply “reflecting on or going back to a past that was” but as “turning it over and over again – iteratively intra-acting, re-diffracting, diffracting anew, in the making of new temporalities (spacetimematterings), new diffraction patterns” (Barad, 2014: 168). The past is thus not something we can re-turn to, as any moment contains traces of “an infinity of moments-places-matterings” (Barad, 2014: 169). In re-turning, Miriam’s child body, childhood, multiple parent bodies and material-discursive parenting practices, and her son’s childhood are opened up, entwined together, and iteratively re-configured, “breathing new life into [them]” (Barad, 2014: 168). Past-present-future child bodies, parent bodies, and material-discursive childhoods and parenting are co-constituting and becoming with and through each other.

As noted by all the mothers in our pilot project, Miriam’s son’s screen-time increased during the pandemic, echoing findings by others (e.g., Findley et al., 2022; Toombs et al., 2022). In the spacetimemattering of the pandemic, Miriam and her partner “like loosen[ed] up about screen time, [tried] not to worry as much.” But Miriam quickly clarified, “like we do still set a lot of limits, and I don’t know if it’s too many…it’s always a work in progress.” In the specific entanglement in Miriam’s story, multiple times are present at once – pandemic time, pre-pandemic time, post-pandemic time, screen-time, and developmental time. The multiplicity of temporality in Miriam’s entanglement stretch time – time in childhood, time in parenting, screen-time, and pandemic time become through their intra-action. “Time” is not one neatly contained or pre-existing entity, instead, is a multiplicity, a collection of forces co-creating entwined materialities like technology and screens, and co-constituting material-discursive practices of parent, child, parenting, and childhood.

Diffracting Miriam’s reflections with Barad’s intra-activity and becoming (2007) illuminates the entwinement of child, childhood, adult, adulthood. Describing parenting with technology, specifically around screen-time and screen use rules as “always a work in progress” foregrounds the intra-activity of parenting, and underscores how various entangled materialities and discourses beyond Miriam’s body and the material-discursive contexts of her family create difference for the iterative becoming of parenting. Entanglements that include various materialities like technologies, multiplicities of past-present-future time, pandemic time, screen-time, and entangled discourses such as age-related technology rules and “good parenting,” intra-act to make legible the material-discursive practices of Miriam’s parenting. Engaging Barad (2007) to decenter Miriam in her story and as the sole actor in her parenting practice, recognizes that parenting is not contained in the flesh boundaries of her body, shaped only by the present, or detached from material-discursive socio-political contexts like the pandemic.

### Diffraction five: Zombie child(hood)-technology–odd parent(hood)

Lana, a graduate student, artist, and mother of a three-year-old boy, self-identified as following a “no screens” parenting approach, except for videocalls with long-distance relatives. Lana shared how her parenting practices around technology seem uncommon, making her feel like “the odd person out” as if she is on an “island where you are like hearing things from other parents… kind of like reacting to your approach.” When asked to elaborate on her parenting approach, Lana said:

Uhm, my mom, [a retired teacher], always talked about how bad screens were for kids, and just her experience with us as kids. […] she talked a lot about how if we watched screens in the morning, we would just kind of turn into zombies […] [My parents] were really specific about when we could watch television […] So I’ve kind of grown-up hearing that. And then I worked in a restaurant, and I would see kids at restaurants with iPads and phones and things, and when [the devices] would be taken away [laughs], [there was] demonstrative behaviour. And then just not having the kids engaging at dinner time or with other people, and [technology] just being used as a soother. And I mean, not to pass judgments, like sometimes that has to be a thing, but I just didn’t want that to be a thing for us, as long as we could.

As Lana said, her mom, her childhood, and “modern day, kids now” informed her approach to parenting with technology. Like Miriam, Lana’s story includes a re-turning (Barad, 2014). Multiple times including Lana’s childhood, her parents’ parenting, observing “modern day, kids now,” intertwine and shape her parenting practices. A figuration of zombie child emerges through a particular intra-action of child-technology-restaurant-parent. The boundaries of zombie child and childhood are necessarily material-discursive. Zombie child body becomes with and through technology, and zombie childhood becomes with and through zombie child body and technology. Similarly, odd parent body and odd parenting become through specific configurations of parent-child-timespace.

Lana’s desire to practice “no screen parenting” for an indeterminate time is marked by the difference that multiple co-existing pasts-presents-futures enact (Barad, 2007). Lana’s past childhood, her more recent past as a restaurant worker, observations of “modern day, kids now,” and her desired present and future childhood for her son become with and through each other. Lana’s parents’ parenting and her childhood are not over, “time is out of joint […] broken apart in different directions” shaping diffraction patterns which affect the possibilities for meaning and experience (Barad, 2014: 169). As Barad (2014) argues, the ‘now’ of Lana’s parenting is not an “infinitesimal slice but an infinitely rich condensed node” that includes a multiplicity of spacetimes – then, there, now.

### Diffraction six: Computer games-age-becoming child-becoming adult

Compared to other mothers we talked to, Jenny had a more regimented approach to screen-time. Jenny explained that each child gets a turn to choose one television clip or video to watch for 10-12 minutes. Rotating from 10–12-minutes turns, their three children could watch up to 32 minutes of television a day, but the caveat was that their siblings might select something they might not like. They maintained this rule throughout the pandemic and started to allow Minecraft or other computer-based educational games. Jenny said, “I think the important thing is to try to find a nice balance […] computers and screens […] they can be good and […] bad depending on what they’re doing with them, and you know, the quality of the programming.” Challenging some of the “best practices” around parenting with technology that recommend no screen-time for children under age two (e.g., Ponti, 2023), Jenny said “I’m okay with kids watching TV like even if they’re aged two, like I think that’s okay.” Jenny also seemed to challenge negative framings of video games, noting “I loved computer games as a kid, I’m okay with my kids playing computer games, but it has to be like, not too much.” Jenny positioned video games as helpful for teaching hand-eye coordination, but she wanted to wait to introduce her children to other video games until “they’re a bit older, so they have more like […] more self-control.” By doing so, Jenny thought “they’ll turn [video games] off without making a big fuss […] [But if you] introduce video games too early on it’ll be harder to establish good habits later.”

Past-present-future parenting affect and are affected by past-present-future childhoods. Multiple spacetimes become in Jenny’s reflections, including developmental time, clock time, and screen-time. With Barad (2017: 57) we might see this multiplicity and intertwinement of various timespaces in the intra-activity of Jenny’s pandemic parenting as indicative of “the urgency to trouble time,” to shift away from understanding time as something that exists prior to intra-actions. Jenny’s weaving of past-present-future remind me that there are traces of past-present-future in any given moment. By exploring patterns of difference and attending to how and when pasts-presents-futures are evoked, we can challenge “beginnings and ends” (Barad, 2017: 75), including around child, childhood, adult, and adulthood.

### Diffraction seven: Gender-age-technology-risky child(hood)

In addition to discussions of the “past,” our interviews also included reflections on how pandemic parenting might shape the “future” of their families’ technology practices. Ruth, for example, seemed worried about the allure of technology as her children aged, especially since the timespace of the pandemic affected screen-time. Ruth told me how she wanted to support her children to become self-regulated technology users. Ruth said, “I want them to understand and be aware of [the effects of technology] so they can make good decisions about their own technology use because I’m not going to be able to police their tech time forever.” Ruth was particularly worried about her son’s new interest in video games, and the potential for social isolation and addiction. Interestingly, several mothers positioned video game addiction as primarily a concern for boys. Diffracting Ruth’s worries with Barad (2007) reveals a specific intra-action of child-childhood-gender-age-parent-parenting-technology. The material-discursive contexts of technology, specific video games, gender, and age intra-act and co-constitute Ruth’s meanings of child and childhood. In this entanglement, the boundaries of child, childhood, youth, and adulthood are open. They are not neatly contained in the sites of material bodies, rather are emergent intra-acting phenomena that become through their collisions in specific spacetimematterings. “Risky” boy child becomes intra-actively with material-discursive video games. “Good” parent becomes intra-actively with entanglements of child-technology-age-gender. In this entanglement, material-discursive contexts like technology diffract with developmental age and gender to co-constitute child bodies and childhood as “at risk” of contamination and tarnish in their becoming with technology.

I was struck by Ruth’s assertion that she cannot “police their tech time forever.” If the self is understood as a multiplicity and superposition of dynamic timespaces (Barad, 2014), then the “future” which Ruth hopes for her children around technology will be marked by particular moments wherein they engaged with technology in the pandemic. Furthermore, their “future” childhoods include traces of their relational becoming with their parents. Ruth’s children’s continual becoming with technology includes traces of the spacetimemattering (Barad, 2007) of the pandemic, including relations of child-adult, child-technology, and adult-technology. Their “futures” with technology are marked by iterative traces of past-present-future childhoods, which are entwined with the past-present-future becoming of Ruth’s childhood and parenting.

I think Ruth is also inviting us to think about the patterns of difference that the pandemic enacts around technology use. It is not possible to simply “move on” from pandemic specific meanings and experiences with technology, or to “turn the clocks back” to pre-pandemic experiences with technology. If we realize that past-present-future are open, porous, multiple, and coexisting, leaving traces in the world’s ongoing intra-activity (Barad, 2017), we must acknowledge that the material-discursive contexts and spacetimemattering of the pandemic will shape past-present-future becomings of parenting, parent, child, and childhood. The “past” then, including the pre-pandemic and pandemic, is not simply something that has left us, instead, it will continue to be felt and entangled with our individual and collective iterations of child and childhood.

## In/conclusions

In the spacetimemattering of this paper, I diffracted seven wondrous (MacLure, 2013) moments from interviews with Barad (2007, 2014, 2017) to encounter how mothers evoke their own childhoods, pre-pandemic childhoods, pandemic childhoods, and post-pandemic childhoods messily and all at once. This paper is sticky as I aimed to complicate binaries of child/adult and linear conceptualizations of time by entwining with data that was co-created through a research apparatus (Barad, 2007) that emerged from humanist and linear framings of child and adult. My ontological approach, inspired by Barad, created difference in the interviews and my engagement with the data. I am acting from within (Barad, 2007). My entangled age and relational becoming child-youth-adult-researcher are differentiating, intra-acting with the research questions, participants, technologies, and thinking that co-create the data and the story that unfolds here. While messy, my re-turn to the data was an enriching practice that pushed me to let go of “an easy sense” (Mazzei, 2014) of the data.

By troubling a linear approach to time (Barad, 2017), the diffractions inspire thinking about how pasts-presents-futures are written through each other. This analysis helps us to think about the boundaries of childhood and adulthood as porous, and recognize child, childhood, parent, and parenting as material-discursive phenomena, always intertwined, iterative, multiple, and becoming with and through each other (Crinall, 2017; Murris, 2016). While I focus on temporality, future agential realist accounts might consider the impossibility of online/offline binaries of child and childhood. There is also a need for more research that deeply analyzes the relations of power like colonialist mineral extraction and environmental degradation in the intra-activity of technology and digital childhoods.

“Troubling time” (Barad, 2017) in Childhood Studies opens possibilities to understand what particular spacetimematterings do for how the material-discursive contexts of child and childhood are constituted, understood, and experienced. An agential realist account (Barad, 2007, 2014, 2017) to understanding the posthuman child (Murris, 2016) decenters the individual child body, to better understand child as an entanglement of bodies, materialities, discourses, and timespaces. Child bodies and adult bodies intra-act and become together, moving beyond a hierarchical and binary conceptualization of child-adult relations. Furthermore, child bodies, childhood, parent bodies, and parenting never finish, as they leave traces in entanglements that affect the iterative unfolding of the world. Through agential realism, we can recognize the relational, intra-acting forces of our own becomings in the ongoing intra-activity of child and childhood. In troubling time, we might better understand the complexity of the particular material-discursive contexts, agencies, and spacetimes that enact difference in the ongoing, multiple, and relational unfoldings of child and childhood.
